# The Relationship between Environmental Information Disclosure and Profitability: A Comparison between Different Disclosure Styles

**DOI:** 10.3390/ijerph16091556

**Published:** 2019-05-03

**Authors:** Hua Yin, Mingyu Li, Yuan Ma, Qiang Zhang

**Affiliations:** College of Economics and Management, Shandong University of Science and Technology, Qingdao 266590, China; sdqdyh@126.com (H.Y.); 17854259419@163.com (M.L.); zhangqiang3637@163.com (Q.Z.)

**Keywords:** environmental information disclosure, profitability, environmental performance, substantive style, symbolic style

## Abstract

Combined with the existing research gap, this paper divides firms’ environmental information disclosure styles into two types: Substantive style and symbolic style. This paper elaborates on the relationship between environmental information disclosure and firms’ profitability of these two disclosure types and tests this relationship using the data from 676 firms employed from the heave-polluting industry. Considering the endogenous and heteroscedasticity problems, 2-stage least squares method and weighted least square method were adopted. The results showed that (1) positive relationships exist between environmental information disclosure and profitability for both types; and (2) the contribution of symbolic-style disclosure to profitability is larger than that of substantive-style disclosure. These findings are important for corporate managers and highlight some policy implications in developing countries.

## 1. Introduction

With the improvement of public environmental awareness, environmental information disclosure (EID) has gradually become an information communication tool commonly used by listed companies [1,2]. It is one of the important initiatives that firms take to fulfill their environmental responsibility and to accept social supervision. Research on the determinants of EID is extensive [3,4,5,6,7,8,9,10,11,12], while research on its consequences is relative scarce [13]. This is precisely the issue that both business managers and policy makers are more concerned about [14].

One research stream in this domain focuses on the investors’ response to EID in the capital market and its impact on corporate value, such as stock price, analysts’ earnings forecasts, and expected future cash flows [13,15,16,17,18,19,20,21,22]. The other stream cares about whether EID would have an impact on business operations, such as financial performance. However, the conclusions from this stream are controversial. Some empirical results show a positive relationship between EID and firms’ financial performance [20,23,24,25,26,27], while others show a negative relationship or nonsignificant relationship [14,28,29,30,31,32]. Differences in environmental legislation and regulation among governments are one of the reasons for the inconsistency of these conclusions [31]. The existing research data mainly come from countries with relatively well-regulated markets, such as Italy, France, Sweden, the United States, and Australia. The empirical evidence from emerging markets is still lacking. Therefore, more extensive evidence is needed to verify the above relationship. In response to this shortcoming, we selected China as the research background. As US President Trump announced that the United States would withdraw from Paris Climate Agreement, China, the world’s second largest economy, will take more important responsibilities in the global environmental improvement.

Although the regulatory authorities have issued guidelines for EID, disclosure is still voluntary. In addition, there is no enforcement and mandatory requirements for disclosure contents [16,19]. In order to maximize firms’ interests, executives usually disclose selectively [10,33,34], so the difference of disclosure style between firms exists [5,12]. Will the disclosure style affect firm performance? Although a few studies have distinguished the contents of environmental information reports [18,20], there is still no further comparison. In response to this research gap, our article classifies the disclosure styles in accordance with the contents of firms’ environmental reports and conducts a comparison by empirical evidence.

The data used in this study are from the heavily polluting industry in China A-Share listed companies. China’s economy has made gratifying achievements in the process of transforming from a centrally controlled planned economy to a market economy, while it has also brought about increasingly prominent environmental problems. In particular, as a main source of environmental pollution, the environmental impact of heavily polluting industry has become an unavoidable important issue for both academia and government regulators. However, since the Chinese government has not formulated specific and clear norms on firms’ EID behavior, firms have more choices in terms of disclosure form, disclosure content, and disclosure quality. This background is in line with the research needs of this paper. The dependent variable in this study is profitability. Profitability is a key resource for the sustainable development of firms and also the most important business indicator that managers care about [35]. Therefore, selecting it as a dependent variable is helpful for guiding management practice.

The objective of this article is twofold. First, we wanted to confirm that EID has a marginal contribution to firms’ profitability. In addition to green walking, green talking is also of value. In order to do this, we controlled environmental performance and considered the endogenous problem that may exist in EID and environmental performance using the 2-stage least squares (2SLS) method. Second, we wanted to verify that information disclosure style also affects firms’ profitability. In order to do this, we identified specific disclosure attributes that help to distinguish firms’ disclosure style.

The potential academic contributions are as follows: (1) Marginal contribution of EID to firms’ profitability was proposed and tested. Environmental performance was added as a control variable, and the endogenous problem was considered. Environmental performance is a major component of firms’ environmental management and has an important impact on firm’s profitability. The lack of environmental performance in prior research may affect the effectiveness of estimates and may even amplify the effects of environmental disclosure. Adding it as a control variable is helpful to test the marginal contribution of EID. Our results show that EID does have a marginal contribution to firms’ profitability. This result could help firms to take reasonable environmental initiatives. (2) A comparison between different disclosure styles was made. Firms are classified into different groups according to the different disclosure styles. The contribution of EID to firms’ profitability was verified, and then a comparison between these two groups was made. The result showed that the contribution of symbolic disclosure to firms’ profitability is higher than that of substantive disclosure, highlighting some policy implications in developing countries.

The rest of this article is organized as follows: Hypotheses are developed in Section 2. Research methods including sample and measurements of the variables are described in Section 3. Results and discussions are depicted in Section 4. Conclusions and implications are in the last section.

## 2. Hypotheses

### 2.1. Classification of Firm’s Information Disclosure Style

In order to attract attention in both the product market and capital market or deal with media pressures, many firms choose to release environmental information on a regular basis. Since EID is voluntary, regulatory authorities do not specify the format and content of the information disclosure [12]. Firms often have their own preferences to release information for different purposes. For example, some firms focus on the publication of data of quantitative results, while some other firms focus on the description of behavioral information [1,36,37]. Therefore, firms can be classified into substantive style and symbolic style based on the contents disclosed in their environmental reports [12,18,20].

### 2.2. The Relationship between EID and Profitability for Substantive-Style Disclosure

Firms that disclose their environmental information in substantive style put emphasis on quantitative data in the information reports. These data always relate to environmental management investments and environmental achievements of their firms. Such an information report is often supported by substantive actions taken by these firms. These actions usually include clean production, pollution control, and green product development. These measures could reduce the environmental hazards generated by the firm [38,39]. Researchers suggest that such substantive actions could help firms to enter specific markets, attain government subsidies, avoid environmental penalties and litigation, etc., thus contributing to the improvement of the firm’s profitability [40,41,42,43,44,45]. Beyond substantive environmental initiatives, could EID create additional value for the company?

Voluntary disclosure theory suggests that information disclosure is an effective tool to reduce information asymmetry [46]. For substantive disclosure firms, information disclosure helps to reduce information asymmetry, thereby reducing agency costs, transaction costs [16], and financial costs [47], optimizing resource allocation, and is conducive to firms which have high environmental quality and strong social responsibility to obtain valuable competitive resources such as financing and market [13,48,49,50]. Therefore, EID could create additional value for these firms. Based on this theory, the study proposes the following hypothesis:

**Hypothesis 1 (H1).**  *EID affects profitability positively for firms with substantive-style disclosure.*

### 2.3. Relationship between EID and Profitability for Symbolic-Style Disclosure

Firms that disclose their environmental information in symbolic style usually focus on their environmental strategy, environmental goals, and environmental protection measures by using a language narrative, while there is a lack of corresponding quantitative information in information reports. Prior studies have pointed out that poorly performing firms tend to disclose environmental information by long reports with complex words and sentences [10,29,34]. The contribution of this type of disclosure to profitability could be analyzed using legitimacy theory. Legitimacy refers to “the actions taken by firms are considered normal, compliant, and commendable in the framework of established social norms, values, and beliefs” [51], being the stakeholders’ perceptions or judgments based on firms’ behaviors [52]. Legitimacy is very important for building a firm’s competitiveness. From the perspective of social legitimacy, the firm’s external environmental propaganda could create a social image of paying attention to the environment and taking social responsibility [8,9,53]. Symbolic disclosure could also cover up the slack of a firm’s environmental initiatives, create a glamorous social image [54,55], and then bring resources to the firm, consolidate relationships with stakeholders, and recruit competitive employees [39]. All of these can ultimately be reflected in the profitability of the firm [13]. Therefore, firms could also seek good profitability in the market through environmental publicity and beautification [39].

Based on the analysis above, the second hypothesis in this study is obtained:

**Hypothesis (H2).**  *EID affects profitability positively for firms with symbolic-style disclosure.*

### 2.4. Comparison of the Contribution of EID to Profitability between Two Disclosure Types

Considering the environmental supervision of the government and society, this study proposes that the contribution of symbolic information disclosure to firm’s profitability is not as good as substantive disclosure. The reasons are as follows:

(1) Excessive descriptive information may affect corporate image. The excessive descriptive information in symbolic disclosure often makes it a smokescreen for poor environmental performance [12,18,20,27]. (2) It would be worse once the public recognizes the truth. Environmental propaganda without actual environmental performance support is more likely to attract media and public attention. When the public discovers this exaggerated behavior, it will lose trust in the firm [55]. In this case, stakeholders tend to stay away from such firms and reduce transactions with them [39]. (3) Exaggeration may cause employees’ antipathy. As insiders of the firm, employees have more perspectives to understand the actual operations of the firm. Therefore, exaggeration is too obvious to employees that leads to their antipathy and conflict, which affects organizational effectiveness and profitability [39].

Combined with the above analysis, the third hypothesis is obtained.

**Hypothesis (H3).**  *The contribution of EID to profitability for substantive-style disclosure is greater than that for symbolic-style disclosure.*

**Figure 1:**
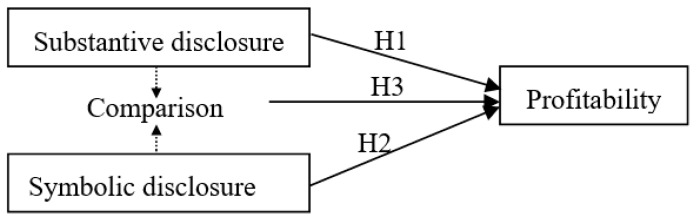
Research framework.

The research framework is depicted in Figure 1.

## 3. Research Design

### 3.1. Variable Measurements

#### 3.1.1. Independent Variable: EID index

Prior research has noted that the information disclosed in a firm’s environmental report can be captured by the extent of the content, such as pages or number of words, and the depth of the content, such as the number of items that are disclosed [10,27]. Using content analysis, which is adopted in relevant research, the disclosed environmental information in firms’ reports is divided into two categories [12,18,56]. One is subjective information, which is usually described in words, typical language such as “developing clean energies”, “keep our homeland beautiful”, and so on. The information conveyed by this type of expression is often vague and not easy to verify. The other one is objective information, which often uses quantitative indicators such as raw material consumption per unit of product, energy consumption, and greenhouse gas emissions. This type of information can accurately convey to stakeholders the environmental efforts and results achieved by the firm. By drawing on the existing research to assign values to these two types of information, the subjective information score is formed by taking the natural logarithm of the number of rows of descriptive language, the objective information score is formed by the number of quantitative information disclosed, and the EID index is obtained by adding them up [12,18,56].

We adopted the two-person independent scoring method in the scoring process. The two raters would conduct the official scoring process only if the intercoder reliability of the trial phase reached 90%. The dispute in the official rating was coordinated by a third person. We performed a reliability test on the final score, and the Cronbach’s α value was above 0.9, indicating that the results are credible.

#### 3.1.2. Dependent Variable: Profitability

Return on equity (ROE) is viewed as an authentic measure of profitability and is widely adopted in literature [1,22,31,36,57]. Considering the causality of EID and profitability in this article, a lagged ROE was employed [36].

#### 3.1.3. Control Variables

The control variables involved in this study are as follows:

Firm size. Studies have shown that firm size can influence its profitability [58]. Larger firms could benefit from scale economy. We take the natural logarithm of the firm’s assets (unit: Million China Yuan) at the end of the year [36].

Slack resources. Slack resources mean that the pool of resources a firm possesses is ample. Agency theorists regard it as a form of waste and an unnecessary cost. Firms with high levels of slack resources may invest in unrelated projects, which would damage profitability [59]. This variable is measured by the firm’s asset-liability ratio [36,39].

Environmental performance. Many studies have proved that environmental performance will affect firms’ profitability [40,41,42,43]. By drawing on existing research, the number of green technology patents of the firm is taken as a proxy variable [31]. We set key words such as “green” or “sustainable development” or “emission reduction” or “energy saving” in the search engine of the Baiteng patent website and finally obtained firms’ green technology patent data. Its natural logarithm was taken to measure environmental performance.

Shareholder concentration. If the largest shareholder has a high proportion of shares, it will have a stronger influence on the firm’s decision-making. Then its effect on profitability will be positive [36,60]. This is measured by the shareholding ratio of the largest shareholder.

Growth. Measured by the growth rate of operating income [36].

Industry. Use the two digits of listed company industry classification number.

The measurements of these variables can be seen in Appendix A.

### 3.2. Identification of Firm’s Disclosure Style

As mentioned earlier, substantive-style of information disclosure tends to highlight quantitative environmental indicators in information reports, while symbolic-style disclosure focuses on a large amount of textual descriptions and nonquantitative information, so we adopted the difference between the objective information score and subjective information score to identify the disclosure style. Since these two scores belong to two dimensions and the information that needs to be disclosed between industries varies, by drawing on the method adopted by Plumlee et al. [20] and Benlemlih et al. [61], we standardized the firm’s objective information score and subjective information score respectively by industry and further calculated their difference. If the result is greater than 0, the quality of the firm’s EID is higher than the quantity, and the disclosure style tends to be substantive. On the opposite, if the result is less than 0, the disclosure style tends to be symbolic.

### 3.3. Research Models

The regression model to be tested is as follows:

(1) $$P_{ij}=\beta_{i0}+\beta_{i1}EIDI+\beta_{i2}SIZE+\beta_{i3}SLA+\beta_{i4}EP+\beta_{i5}CON+\beta_{i6}IND+\beta_{i7}GRO+\varepsilon_{ij}$$

(2) $$P_{j}=\beta_{0}+\beta_{1}EIDI+\beta_{2}EIDI\times DS+\beta_{3}SIZE+\beta_{4}SLA+\beta_{5}EP+\beta_{6}CON+\beta_{7}IND+\beta_{8}GRO+\varepsilon_{j}$$

In Models (1) and (2), P is a dependent variable, which means the firm’s profitability. EIDI is an independent variable, which means EID index. SIZE, SLA, EP, CON, IND, GRO are control variables, representing firm size, slack resources, environmental performance, shareholder concentration, industry, and growth, respectively. ε is random error. The value of i in Model (1) is either 0 or 1. i = 1 stands for a firm with a substantive style. In this case, Model (1) corresponds to hypothesis 1. If β_11_ > 0, hypothesis 1 is supported. i = 0 stands for a firm with symbolic style, and in this case, Model (1) corresponds to hypothesis 2. If β_01_ > 0, hypothesis 2 is supported. j = 1, 2, 3, …, n represents the j-th sample firm with the i-th disclosure style.

In order to test hypothesis 3, it is necessary to compare β_01_ and β_11_. Using Chow’s test [62], we created a regression model, as shown in Model (2), adding a new variable, named DS, to indicate the disclosure style (i = 1, is substantive style; i = 0, is symbolic style), and merging the two samples in Model (1). If β_2_ in Model (2) is greater than 0, hypothesis 3 is supported.

### 3.4. Sample Selection

The research samples in this paper were taken from China’s A-share listed companies which were in the heavily polluting industry from 2015 to 2016. The heavily polluting industry was selected based on the following considerations: (1) It is a frequent concern in relevant studies. For example, Walker and Wan [24] used data from 103 Canadian firms in heavy polluting industries such as chemicals, energy, and mining. Clarkson et al. [18] made their research via environmentally sensitive industries. (2) Government regulation and media attention are relatively high for the heavily polluting industry, which is in line with the preset conditions in the third hypothesis of this study. We selected the year 2015 as the beginning of the sample period because the environmental regulation and enforcement in China have been stringent since 2015 [36]. The end of the sample period is 2016, since the latest available variable of the lagged profitability was in 2017.

According to the “Guidelines on Industry Classification of Listed Companies (revised in 2012)” issued by the China Securities Regulatory Commission (CSRC) and the classification of heavily polluting industries stipulated in “the List of Classified Management of Environmental Protection Verification Industry of Listed Companies (2008)” published by the Environmental Protection Administration (EPA), this paper collected the listed companies from 22 industries, including no-ferrous metal smelting and rolling processing industries, pharmaceutical industry and textile industry, etc. The independent variable in the research was obtained by content analysis of the environmental disclosure information in the corporate social responsibility reports. The control variables were taken from the financial data in the annual report of the listed companies, and the sources were from the WIND and the CSMAR database. In addition, firms’ green patent data were reviewed from the Baiteng Patent Database. Considered the lagged effect of the impact of EID on profitability, the data for the dependent variable were obtained from the firms’ annual reports of 2016–2017. In the end, firms with incomplete data were eliminated, and a total of 676 valid year-company observations were obtained.

## 4. Results

### 4.1. Descriptive Analysis and Correlations

Combined with the identification method of disclosure style, 676 sample firms were divided into two subsamples, among which 312 firms belonged to substantive style and 364 firms to symbolic style. This result is comparable to that of Fernando et al. [63]. More than a half of the sample firms were classified as greenwashers in their research. Descriptive statistics of the two subsamples are shown in Table 1 and Table 2, respectively. Further independent t-tests showed significant differences between the two groups of samples in firms’ profitability, EID, and environmental performance. The profitability and environmental performance of substantive-style firms are better than those of symbolic-style firms.

In order to verify the validity of the classification results, we compared the information reports of the sample firms in mining industry. Some of the sample firms were classified as substantive-style, including Yanzhou Coal Mining Co., China Shenhua Energy Co. and so on. In these firms’ environmental information reports, there were not only the firm’s environmental mission, environmental organization structure, environmental management measures, etc., but also the accurate quantitative data on environmental protection investment, energy-saving investment, comprehensive energy consumption, energy consumption per unit of output, logistics energy consumption, water consumption, wastewater utilization, pollutant gas emissions (SO_2_, NO_x_), land reclamation, etc. The symbolic-style firms included Zijin Mining Co., Tibet Mineral Co. and so on. Although the information on pollutants such as wastewater and waste gas was disclosed in the information report of Zijin Mining Co., the focus of the report was the national environmental standards that it complies with and the environmental certification it adopts. The typical statements used in its report are as follows: “strictly abide by the ‘Water Law of the People’s Republic of China’ and other legal standards” and “effectively strengthen the organization”, and the information provided was not specific compared to China Shenhua. In the environmental information of Tibet Mineral Co., there is no disclosure of the quantitative information: It only provides the firm’s management measures in terms of rules and regulations and employee training. A detailed comparison about the contents of these two disclosure styles can be seen in Appendix B. By comparison, it is shown that the information report of symbolic style focuses on vague information such as company system, management process, and environmental standards the firm complies with.

### 4.2. Regression Results

Taking into account the endogenous problems that may exist in the EID and environmental performance, the 2SLS method was adopted in all of our models. In the first step, the EID index was used as a dependent variable, and environmental performance was used as an independent variable for regression, then the residual of dependent variable and regression value were calculated. The second step was to use the residual as an instrumental variable of the EID index and involve it in subsequent calculations.

#### 4.2.1. Regression Results of Substantive-Style Firms

To test hypothesis 1, the ordinary least squares method was employed initially. Control variables were used in Model 1, and independent variable EID index was added in Model 2. As the results show in Table 3, the coefficients are insignificant. Considering the possible heteroscedastic problem, we used the White test, and the result indicated that the heteroscedastic problem exists (*p* value is 0.000). Therefore, the weighted least squares method was adopted to correct the model. Taking the result in Model 2 and using it to calculate the regression value to obtain residual, and using the reciprocal of the squared residual term as the weight to perform the weighted least squares, finally, the adjusted Model 2 was obtained. It can be seen from the results that the coefficient of the EID index on the firm’s profitability is 0.149, which is positive and significant, so hypothesis 1 is verified.

#### 4.2.2. Regression Results of Symbolic-Style Firms

To test hypothesis 2, the ordinary least squares method was adopted in Model 3 and Model 4 initially. The independent variables of Model 3 were control variables, and an independent variable was added to Model 4. The relationship between the main variables and profitability was not significant, and the F value of the whole model was not significant either. Considering the heteroscedastic problem, the white test was adopted, and the null hypothesis was rejected, indicating the existence of the heteroscedastic problem. Based on Model 4, the weighted least squares method was adopted, and the adjusted Model 4 was obtained. The result showed that the F value of the whole model is 16.093, the adjusted *R*^2^ is 0.773, which is obviously better than Model 4. The EID index has a positive effect on the firm’s profitability (β = 0.108, *p* < 0.05), so hypothesis 2 is verified.

#### 4.2.3. Comparison

To test hypothesis 3, we combined the two samples above into one. Model 5 shows the regression result of the control variables. Model 6 adds the independent variable EID index and its interaction with the disclosure style. Since the results of the entire model are not significant, the White test and corresponding data processing were further performed. The adjusted results showed that the coefficient of the interaction term is −0.255, which passes the significance level of 0.01, indicating that the two types of EID have different contributions to the firm’s profitability. Since the coefficient is negative and the reference group in the study is symbolic-style disclosure, this result indicates that the marginal contribution of symbolic-style disclosure to the firm’s profitability is greater than substantive-style disclosure, which is exactly the opposite of our expectation. Therefore, hypothesis 3 is not verified.

### 4.3. Robustness Test

In order to verify the robustness of the results, we took the following measures: (1) Replacing the dependent variable with earnings per share and recalculating; and (2) taking 3 times the standard deviation of the independent variable to eliminate the outliers in the sample, then conducting a recalculation. After taking the above measures, the operation results of main variables had not changed.

### 4.4. Discussions

The positive relationship between EID and profitability for substantive-style firms could be explained by agency theory. Firms that conduct substantive disclosure usually have actual environmental practices as a foundation, and these environmental behaviors could improve the firm’s profitability theoretically. However, considering the information asymmetry, firms’ environmental practices would not be accurately delivered to external stakeholders, which makes it difficult for firms to take credit for it. EID would be helpful to make up for the above shortcomings. Therefore, firms could obtain more resources and opportunities in the market, so as to achieve better profitability.

Symbolic-style disclosure firms put more effort into describing behaviors and corporate visions in their information reports. Although this style of disclosure lacks solid environmental achievement as support, it could help the firm to build an environmentally responsible image in the market, seek social legitimacy, and lobby stakeholders in the capital market, product market, and other fields to obtain rare resources.

However, the comparison between the two types gives us a surprising result in that symbolic-style disclosure has contributed more to a firm’s profitability. According to this result, we conducted an analysis from the perspective of cost and income.

The disclosure costs of the two styles are comparable under the implementation of current environmental regulations in China. Heavily-polluting firms have always been the focus of Chinese government monitoring. Environmental protection authorities carry out online monitoring of such firms’ waste water, exhaust gas, and other major pollutants. Firms are required to report data on energy consumption and major pollutant emissions to the local authorities on a monthly basis. Therefore, adding quantitative data to environmental information reports will not bring too much extra cost to them. Under such a circumstance, firms with good environmental performance prefer to provide some hard information in their information reports. Poor environmental performers prefer to disclose less quantitative data or not to disclose them at all [10,29,34].

However, exaggerated symbolic disclosures have created more benefits for firms due to public reading habits and the absence of disclosure regulation. (1) Public reading habits. The public lacks the expertise of environmental practices and may prefer symbolic textual information when reading environmental reports, so textual disclosure could be more impressive [24]. (2) The absence of disclosure regulation. Audit is not necessary, since EID is voluntary. The publicity and beautification in environmental information will not be uncovered by outsiders; thus, the public will be blinded, and the market resource allocation will be out of order.

## 5. Implications and Limitations

### 5.1. Implications

Combining the research conclusions, we could give implications to both academia and practitioners, including corporate managers and regulators.

#### 5.1.1. Implication for Academia

Regarding the academia implication, our result shows a more significant positive relationship between symbolic-style disclosure and firms’ profitability. To the best of our knowledge, few studies distinguish the styles of information disclosure in the existing research of EID–performance relations except Plumlee et al. [20]. Plumlee and his co-authors found that soft disclosure relates to the firm’s market performance positively. A comparison in our study provides more empirical evidence in this domain. These results showed that both EID and the disclosure style will affect firms’ financial performance.

#### 5.1.2. Implication for Managers

This research can provide relevant insights for business managers. Environmental performance as a control variable shows a positive effect on firms’ profitability in the model. It illustrates that China has initially formed a market environment suitable for sustainable development, and substantial efforts in environmental protection could benefit firms. Entrepreneurs and business decision-makers should actively explore environmental practices while developing the economy. In addition, removing the influence of environmental performance on the firm’s profitability, EID itself also has a marginal contribution to the firm’s profitability. Firms need to protect the environment actively; meanwhile, they should pay attention to corporate propaganda, which is also crucial.

#### 5.1.3. Implication for Regulators

This study also provides implication for environmental disclosure norms. Although China has initially formed a suitable market environment for green development, the two disclosure styles under the existing market supervision have different contributions to firms’ profitability. Symbolic-style disclosure contributes more. On one hand, this may be attributed to the reading habits of stakeholders. On the other hand, it is also due to the lack of information disclosure norms, leaving discretional space for firms. Although the relevant departments have issued a series of guidance documents over the years, there is still an operating space in the format and content details of EID. Firms have a wide selectivity. Therefore, the government should improve the information disclosure norms as soon as possible. By adopting disclosure norms, firms under key monitoring will deliver true and reliable information.

There is also a need for improvement in the supervision of the disclosed information. There are two regulators involved in environmental issues of China’s listed companies. One is EPA, which regulates the environmental performance of listed companies. The other is CSRC, which supervises information disclosure. The decoupling of regulation and supervision leaves space for firms’ symbolic and exaggerated information disclosure. It is very important to establish the information sharing and collaboration mechanism and strengthen the joint supervision of EID of listed companies.

The certification of environmental information by an independent third party is needed. Listed companies always selectively disclose environmental information in order to earn more profits, thus affecting authenticity. For most of the market participants, the lack of expertise makes it hard for them to assess the firm’s environmental responsibility accurately. The certification of environmental information by a third party could ensure the reliability of environmental information to a certain extent and objectively improve the quality of environmental information. Additionally, environmental quality assessment and media supervision are also necessary. They could increase the firm’s cost of environmental information fraud and strive to correct the market’s wrong reaction.

### 5.2. Limitations and Future Research Directions

This article empirically analyzed the relationship between EID and firms’ profitability on the basis of a clarification of disclosure style. The data used in this research are heavy-polluting industries from Chinese listed companies. Data from only one country may influence the conclusion’s universality. In order to test their robustness, data from other countries is necessary. Information disclosure style can affect firms’ profitability. A more detailed research can be made in the future, focusing on details such as the linguistic style, word expression, and readability of the environmental report.

## 6. Conclusions

This study analyzed the relationship between EID and firms’ profitability. In order to have a deep understanding of the impact of EID on profitability, we further divided our sample into two groups in accordance with the firm’s disclosure style. We drew the following conclusions: (1) Through the empirical analysis of 312 substantive-style disclosure firms, the result showed that EID is valuable itself. (2) The analysis of 364 symbolic-style firms showed that their EID also has a positive impact on firms’ profitability. (3) The comparison between these types indicates that the contribution of EID to profitability for symbolic-style firms is higher than that for substantive-style firms.

## Figures and Tables

**Table 1:** Descriptive statistics of substantive-style firms.

	Mean	S.D.	SIZE	SLA	EP	CON	GRO	EIDI
SIZE	4.099	0.620	1.000					
SLA	0.430	0.187	0.506 **	1.000				
EP	1.857	1.571	0.510 **	0.259	1.000			
CON	0.368	0.134	0.379 **	0.152	0.387 **	1.000		
GRO	0.112	0.543	0.142 *	0.112	0.134	0.231**	1.000	
EIDI	4.411	0.815	0.426 **	0.240	0.427 ***	0.493**	0.054	1.000
P	0.139	0.138	0.023	−0.033	0.171 *	0.142*	0.215 ***	0.121 **

*** *p* < 0.01; ** *p* < 0.05; * *p* < 0.1. S.D. = standard deviation; SIZE = firm size; SLA = slack resources; EP = environmental performance; CON = shareholder concentration; GRO = growth; EIDI = environmental information disclosure index; P = profitability.

**Table 2:** Descriptive statistics of symbolic-style firms.

	Mean	S.D.	SIZE	SLA	EP	CON	GRO	EIDI
SIZE	4.271	0.553	1.000					
SLA	0.503	0.196	0.502 **	1.000				
EP	1.046	1.173	0.341 **	0.048	1.000			
CON	0.367	0.164	0.418 **	0.206	0.044	1.000		
GRO	0.133	0.575	0.155 *	0.103	0.221	0.274 *	1.000	
EIDI	3.792	0.667	0.530 **	0.236 *	0.393 ***	0.329 **	0.85	1.000
P	0.114	0.109	0.076	−0.012	0.061 *	0.278 *	0.264 ***	0.177 **

*** *p* < 0.01; ** *p* < 0.05; * *p* < 0.1. S.D. = standard deviation; SIZE = firm size; SLA = slack resources; EP = environmental performance; CON = shareholder concentration; GRO = growth; EIDI = environmental information disclosure index; P = profitability.

**Table 3:** Regression results.

	Model 1	Model 2	Adjusted Model 2	Model 3	Model 4	Adjusted Model 4	Model 5	Model 6	Adjusted Model 6
SIZE	−0.086	−0.084	0.248	−0.047	−0.065	−0.066	−0.081	−0.077	−0.269 ***
SLA	−0.051	−0.050	−0.033	−0.054	−0.054	−0.324 **	−0.051	−0.049	−0.200 ***
EP	0.185	0.183	0.379 **	0.066	0.073	0.185 **	0.141	0.139	0.340 ***
CON	0.111	0.115	0.282 **	0.306 **	0.298 **	0.278 ***	0.222 **	0.227 **	0.705 ***
GRO	0.117	0.119	0.338 ***	0.175	0.215	0.235 ***	0.204	0.215	267 ***.
IND	0.288	0.194	0.139 *	0.336	0.147	0.168 *	0.366	0.232	0.190 *
EIDI		0.110	0.149 ***		0.045	0.108 **		0.031	0.178 **
inter								−0.068	−0.255 ***
*R^2^*	0.217	0.219	0.748	0.186	0.188	0.779	0.067	0.069	0.873
Adjusted *R^2^*	0.165	0.166	0.664	0.143	0.144	0.773	0.033	0.017	0.866
F	0.583	0.457	9.39 ***	1.318	1.572	16.093 ***	1.942 **	1.314	121.804 ***

*** *p* < 0.01; ** *p* < 0.05; * *p* < 0.1. SIZE = firm size; SLA = slack resources; EP = environmental performance; CON = shareholder concentration; GRO = growth; IND = industry; EIDI = environmental information disclosure index; inter = environmental information disclosure index × disclosure style. The dependent variable is profitability.

## Appendix A. The Measurement of the Main Variables

**Variables**	**Explanations**
Profitability	Return on equity.
EID index	Take the sum of subjective information score and objective information score.
Firm size	Take the natural logarithm of the firm’s assets.
Slack resources	Measured by the firm’s asset-liability ratio.
Environmental performance	Natural logarithm is taken after the number of green technology patents plus 1.
Shareholder concentration	The shareholding ratio of the largest shareholder.
Growth	The growth rate of operating income.
Industry	Use the two digits of listed company industry classification number.
Disclosure style	=1, if it belongs to substantive style; =0, otherwise.

## Appendix B. A Comparison of the Disclosure Contents between the Two Styles

	**The content of Disclosure**	**Substantive Style**	**Symbolic Style**
Subjective information	Environmental objective	always	always
	Organizational structure	always	always
	Staff training	often	often
	Environmental impact assessment	often	seldom
	Environmental certification	seldom	often
	Environmental standards	seldom	often
	Environmental policy	often	always
	Environmental auditing	seldom	seldom
Objective information	Land remediation	often	seldom
	Effluent discharge	always	seldom
	GHG emission	always	seldom
	Waste discharge	often	seldom
	Energy consumption	always	often
	Energy saving	always	seldom
	Environmental investment	often	often
